# Factors influencing medication adherence in co-morbid hypertension and diabetes patients: A scoping review

**DOI:** 10.1016/j.rcsop.2024.100426

**Published:** 2024-02-29

**Authors:** Adwoa Oforiwaa Kwakye, Irene A. Kretchy, Prince Peprah, Kofi Boamah Mensah

**Affiliations:** a Department of Pharmacy Practice and Clinical Pharmacy, School of Pharmacy, College of Health Sciences, University of Ghana, P. O. Box LG 43, Legon, Ghana; b Department of Geography and Rural Development, Kwame Nkrumah University of Science and Technology, Kumasi, Ghana; c Department of Pharmacy Practice, Faculty of Pharmacy and Pharmaceutical Sciences, College of Health Sciences, Kwame Nkrumah University of Science and Technology, Kumasi, Ghana

**Keywords:** Medication adherence comorbidity hypertension diabetes

## Abstract

**Introduction:**

Interest in medication adherence has expanded significantly, especially in relation to the management of hypertension or diabetes in recent years. A scoping review that focuses on medication adherence in the co-morbidity of hypertension and diabetes provides crucial guidance for effective management of these interrelated diseases.

**Aim:**

To conduct a scoping review of factors associated with medication adherence among individuals with co-morbid hypertension and diabetes.

**Methods:**

The evaluation was conducted in accordance with the PRISMA-ScR guidelines to ensure the quality of the study. We searched three databases (Scopus, CINAHL, Medline) and one search engine (Google Scholar) from April 2023 to July 2023 on studies related to medication adherence in co-morbid hypertension and diabetes. Except for reviews there were no restrictions on design, location, and time of study.

**Results:**

In total, 972 studies that were not duplicated were obtained. After eligibility and screening procedures were completed, 31 articles were ultimately included in the scoping review. Medication adherence was significantly affected by patient, condition, therapy, socio-economic and health related factors. Intervention trials revealed that education and counselling by pharmacists, nurses, physicians, diabetes educators, community health workers and the use of telephone to motivate patients significantly improved medication adherence.

**Conclusion:**

This review shows the intricate factors influencing medication adherence in patients with co-morbid hypertension and diabetes, emphasizing the need for tailored interventions involving healthcare professionals, policymakers, and researchers.

## 1 Introduction

The co-occurrence of hypertension and diabetes is a serious public health burden.^1^^,^^2^ Globally, co-morbidity is alarmingly prevalent, with statistics estimating that over 40% of patients with diabetes also suffer from hypertension.^1^^,^^3^ This dual burden is especially apparent in older persons, underlining the challenges posed by an aging population.^4^^,^^5^ These chronic diseases are related with an increased risk of cardiovascular disease, renal failure, stroke, and high mortality.^1^^,^^2^^,^^6^ Diabetes and hypertension co-occurrence increases cardiovascular risk synergistically.^1^^,^^7^ Underlying the mechanisms of this connection are intricate networks of vascular dysfunction, insulin resistance, inflammation, and oxidative stress. Co-morbid patients are more likely to develop atherosclerosis, endothelial dysfunction, and left ventricular hypertrophy, which increases their susceptibility to adverse cardiac events^8^^,^^9^. Co-morbid hypertension and diabetes have substantial economic and societal implications that transcend individual health.^2^^,^^10^ The need for medical services, hospitalizations, and long-term care has soared because of these co-morbidities. The financial burden is exacerbated by the need for sophisticated treatment regimens, frequent physician visits, and high medications costs.^10^^,^^11^

Hypertension and diabetes co-morbidity present various hurdles that negatively impact medication adherence.^12^^,^^13^ The necessity of managing two chronic diseases imposes additional cognitive and logistical pressures on patients, resulting in frequent medication nonadherence.^13^^,^^14^ Controlling blood pressure and blood glucose simultaneously can result in regimens that are so complex as to be overwhelming for patients. The progression of a disease and its prognosis are significantly impacted by drug adherence. In co-morbid patients, poor adherence can raise the risk of cardiovascular events, renal issues, and microvascular damage. Nonadherence disrupts blood pressure and glucose regulation, reducing the therapeutic efficacy of medications and increasing the chance of side effects.^15^^,^^16^This exposes patient to increased symptom burden and diminished quality of life.^17^^,^^18^

Medication adherence not only affects physiological parameters, but also improves patients' overall quality of life. Blood pressure and glucose levels that are well-managed contribute to reduced symptoms, enhanced energy, and improved mental health.^19^^,^^20^

Medication adherence is a pillar of effective illness treatment in the complex context of hypertension and diabetes co-morbidity.^16^^,^^21^ The interaction between these diseases needs a rigorous and thorough treatment strategy, with the patient bearing responsibility for drug adherence. A scoping review concentrating on medication adherence in hypertension and diabetes co-morbidity serves as a key compass for navigating the intricacies of these intertwined illnesses.

## 2 Methods

### 2.1 Study design

A scoping review was done using the methodological approach proposed by Arskey and O'Malley and advanced by Levac et al.^22^ The six-step methodology included: a) identification of research questions; which is typically broad in scope b) identification of relevant research articles; c) selection of studies; including the development of criteria for inclusion and exclusion, predicated on a competence in the field of literature d) data charting and synthesis; involving the sifting, sorting, and charting of information in accordance with significant issues and themes e) summary, discussion, and analysis; this stage yields a thematic analysis and a descriptive and numerical summary of the data; and f) discussions with stakeholders; Is a consultation exercise, which involves key stakeholders in order to validate and inform the study findings. Since this review did not involve external stakeholders, the sixth phase, which included consultations with stakeholders, was not utilized. The scoping review was reported using the Preferred Reporting Items for Systematic reviews and Meta-Analyses extension for Scoping Reviews (PRISMA-ScR). The research team drafted the protocol (AOK and IAK). All steps of study selection were conducted by two independent reviewers, with consensus meetings serving as tiebreakers to ensure that all eligibility criteria were properly applied.

### 2.2 Identification of research questions

The primary objectives were to identify factors related to medication adherence, instruments for evaluating adherence, categorize strategies for promoting medication adherence, and assess their outcomes. This process was guided by the following research questions: 1. What are the key factors that influence medication adherence in patients with co-morbid hypertension and diabetes? 2. Which measures are used in the evaluation of medication adherence in patients with co-morbid hypertension and diabetes? 3. How can medication adherence strategies be categorized based on their approach or intervention type in comorbid hypertension and diabetes? 4. What are the outcomes associated with various strategies employed to promote medication adherence?

### 2.3 Identification of relevant research articles

From April to July 2023, three databases (Scopus, Medline and CINAHL) and one search engine (Google Scholar) were searched. This review was based on studies conducted on medication adherence in comorbid hypertension and diabetes with no restriction on location and time of study. The search terms used four main keywords: “comorbidity”, “medication adherence” and “hypertension and diabetes”. They were combined using Boolean Operator (AND, OR, NOT). Medical subject subheading (MeSH) terms were used for the search in Scopus, Medline, and CINAHL. The final search terms used in SCOPUS were as follows. “Medication AND adherence”, “comorbidity OR multimorbidity”, “hypertension AND diabetes”, In MEDLINE MH “Medication Adherence+”) OR (MH “Patient Compliance+”), (MH “Comorbidity+”) OR “comorbidity” OR (MH “Multimorbidity”), “Hypertension and diabetes” OR (MH “Diabetes Mellitus+”) OR (MH “Diabetes Mellitus, Type 2+”) and in CINAHL “medication adherence,” “comorbidity” or “multimorbidity” “hypertension and diabetes”.

Inclusion criteria involved documents that were 1) authored in English, 2) peer-reviewed and literature-indexed to ensure quality, 3) concentrated on patients with hypertension and diabetes co-morbidities and 4) presented systematically with well-described medication adherence measures. Studies that did not have an abstract during the screening phase or the full-text version during the eligibility step (after direct contact with the authors) were excluded. There were no research type or design-based exclusions except for reviews.

### 2.4 Selection of studies

**Fig. 1:**
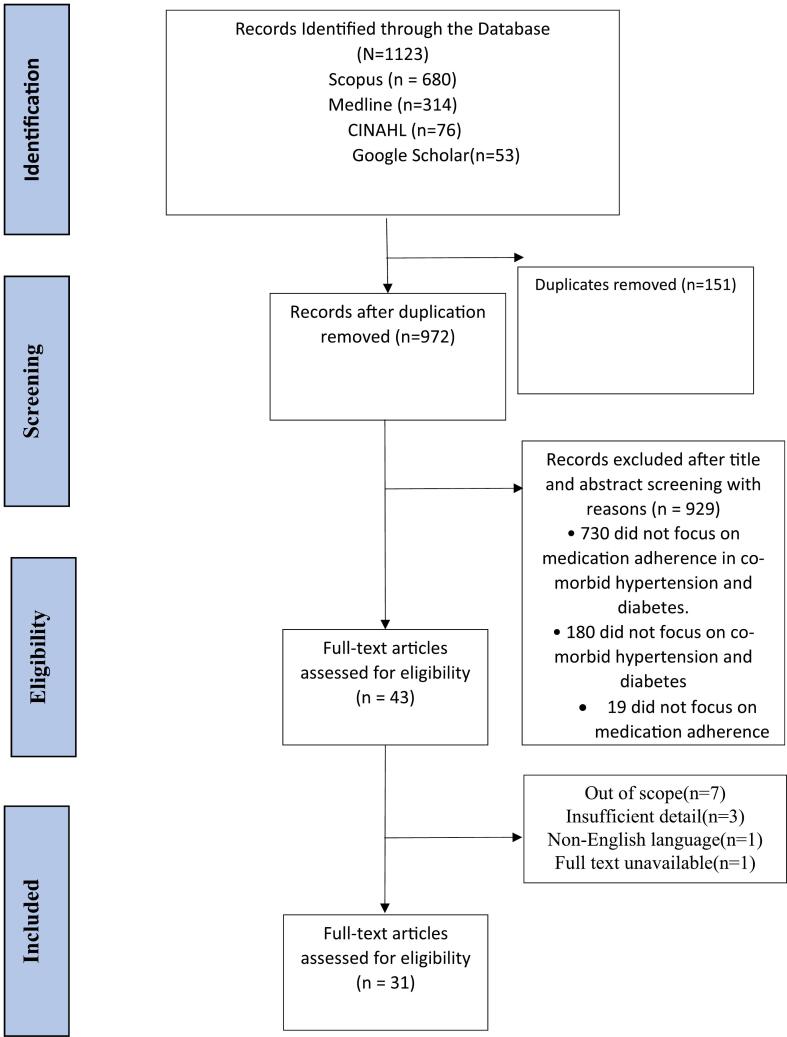
Study selection flow chart.

Two members of the research team (AOK and IAK) evaluated the eligibility of each paper independently. The evaluation was conducted procedurally with a screening of the title and abstract followed by a screening of the whole text (Fig. 1). Disagreements over the eligibility of an article for inclusion in the review were resolved through discussion.

### 2.5 Quality assessment of the included studies

Two researchers (AOK, IAK) assessed the quality of the included studies independently using the applicable critical evaluation checklists from the Joanna Briggs Institute(JBI) to evaluate the methodological quality of the eligible studies. When there were discrepancies, two other reviewers were consulted (PP, KB). Four quality assessment methods from the Joanna Briggs Institute Tools were utilized for randomized control trial, cross-sectional analytical research, cohort studies and qualitative investigations Table 1. For the randomized control trial assessment, thirteen domains were evaluated as “Yes (present), No (absent), Unclear (insufficient information), or Not Applicable.” The cross-sectional assessment of analytical quality, eight domains were evaluated as “Yes (present), No (absent), Unclear (insufficient information), or Not Applicable.” The qualitative studies checklist consists of ten dimensions scored as “Yes” (present), “No” (missing), “Unclear” (insufficient information), or “Not Applicable”. Similarly, the checklist for cohort studies consists of eleven items that are scored as Yes (present), No (absent), Unclear (insufficient data), or Not Applicable. The checklist criteria were not altered, but they were interpreted in a flexible manner to reflect our emphasis on the medication adherence in co-morbid hypertension and diabetes patients.

**Table 1:** Summary of studies of medication adherence in patients with co-morbid hypertension and diabetes.

Study	Title	Country	Study type	Type of measure	Adherence Measuring tools	Level of Adherence	Interventions made	Study recommendations to improve adherence	Quality assessment JBI Synthesis and checklist %
^26^	A Pharmacist Telephone Intervention to Identify Adherence Barriers and Improve Adherence Among Nonadherent Patients with Comorbid Hypertension and Diabetes in a Medicare Advantage Plan	United states of America	A retrospective cohort study	proportion of days covered (PDC)	PDC (proportion of days covered)	80%	Pharmacist Brief telephone intervention	Incorporating MI techniques with follow-up calls to address adherence barriers may be more influential in forming sustainable behavioral change and enhancing medication adherence	54.5
^35^	Pattern and explanatory factors for medication adherence among patients with hypertension, diabetes mellitus and their comorbidity in a north central state of Nigeria	Nigeria	Cross-sectional study	Self-report	Morisky Medication Adherence Scale	49.20%		This study recommends strategies to reduce multiple drug combinations and promote medication adherence counselling and education among patients.	75
^47^	Social Support, Treatment Adherence and Outcome among Hypertensive and Type 2 Diabetes Patients in Ambulatory Care Settings in southwestern Nigeria	Nigeria	Cross-sectional study	Self-report	Morisky Medication Adherence Scale	N/A	N/A	The need for expanded social support system to consistently ensure improved therapeutic outcome among patients.	75
^36^	Adherence and quality of life among diabetic patients with hypertension	Indonesia	cross-sectional study	Self-report	(Modified-Adherence Questionnaire (MAQ	23.70%	N/A	We suggest giving intervention for improving patient's adherence is necessary so the therapeutic targets can be achieved and patient's quality of life can be improved with the DM and hypertension therapy.	75
^24^	Multiple Medication Adherence and its Effect on Clinical Outcomes Among Patients With Comorbid Type 2 Diabetes and Hypertension	United states of America	Retrospective observational study	administrative claims and electronic medical records	PDC (proportion of days covered)	24.80%	N/A	Patients managed by physicians who prescribed statin more often, and patients received care from the same physician for both diseases.	100
^17^	Factors influencing long-term medication non-adherence among diabetes and hypertensive patients in Ghana: A qualitative investigation	Ghana	In-dept interviews	N/A	Interview(survey)			Policy makers to act swiftly with appropriate interventions to encourage adherence among patients.	70
^16^	Medicated-related burden and adherence in patients with co-morbid type 2 diabetes mellitus and hypertension	Ghana	cross-sectional study	Self-report	MARS 5	36.80%	N/A	The interventions should also encourage fixed-dose drug combination to reduce medication-related burden while promoting better adherence and clinical outcomes.	87.5
^25^	Impact of pharmaceutical education on medication adherence and its clinical efficiency in patients with type 2 diabetes and systemic arterial hypertension	Mexico	Randomized control trial	Self-report	Morisky Medication Adherence Scale	Intervention group (43.5%) and the control group (2.3%)	Education and counselling by pharmacist	This highlights the need for patient-centered medical care to include pharmacist-delivered educational and counselling strategies.	84.6
^33^	Medication adherence and direct treatment cost among diabetes patients attending a tertiary healthcare facility in Ogbomosho, Nigeria.	Nigeria	Cross-sectional study	Self-report	Morisky Medication Adherence Scale (MMAS-8)	40.30%	N/A	There is a need for the integration of generic medicines into routine care as a way of further reducing the burden of healthcare expenditure on the patients.	75
^40^	Influential factors in adherence to the therapeutic regime in patients with type 2 diabetes and hypertension	Poland	Cross-sectional study	Self-report and adherence to Refills.	The Adherence to Refills and Medications Scale	59%	N/A	Patients with co-existing hypertension and diabetes require precise, tailored health actions.	75
^42^	Effects of Emotional Response on Adherence to antihypertensive medication and blood Pressure Improvement	United states of America	Cross-sectional study	Pharmacy prescription refills	Refill compliance, Percentage of days covered	50.8% (males), 57.8%(Females)	N/A	future studies would further validate ER(emotional response) and evaluate these initial descriptions of effects of ER on adherence to blood pressure medication and on blood pressure improvement.	75
^45^	Association between medication adherence and quality of life of patients with diabetes and hypertension attending primary care clinics: a cross-sectional survey	Saudi Arabia	cross-sectional study	Self-report	Morisky Medication Adherence Scale(Atinga et al., 2018)	21.70%	N/A	It is critical for healthcare professionals engaged in providing care to patients with diabetes and/or hypertension to involve patients in decision-making process	75
^21^	Clinical pharmacists' education and counselling in patients with co-morbid hypertension and diabetes in a Municipal hospital in Ghana	Ghana	Intervention study	Self-report	MARS 10	Month 3(30.47 vs 19.23%, p < 0.0001) and month 6(39.64 vs 22.19%, *p* < 0.0001	Clinical Pharmacist education and counselling	Clinical pharmacy services should be instituted at the hospitals	84.6
^14^	Association of belief about medication on drug adherence for the treatment of type 2 diabetes mellitus, hyperlipidaemia and hypertension in the community of two selangor districts	Malaysia	Cross-sectional study	Self-report	ARMS(Adherence to Refills and Medication Scale)	N/A	N/A	The findings suggest that patients' beliefs about medication, including medication concern, necessity and harm could have a limited influence on patients' adherence to their medication.	75
^48^	Knowledge, attitudes, and adherence to treatment in individuals with hypertension and diabetes mellitus	Brazil	Cross-sectional study	Self report	Martín-Bayarre-Grade(MBG)	N/A	N/A	Adherence to treatment implies an active attitude with spontaneous and collaborative involvement of the health professional and patient in a process of reciprocity, which leads to behavior change	75
^46^	Medication adherence and determinants of non-adherence among south Indian diabetes patients	India	Cross-sectional study	Self-report	Medication Adherence Questionnaire(MAQ)	55.80%	N/A	Medical community needs health professionals to educate the patients about their disease states and compliance to prescribed medications.	50
^27^	A Motivational Interviewing Intervention to Improve Adherence to ACEIs/ARBs among Nonadherent Older Adults with Comorbid Hypertension and Diabetes	United states of America	Intervention study	Proportion of days covered	Intervention = PDC (proportion of days covered)	N/A	Telephone motivational intervention	This reveals that a brief telephonic MI intervention may be effective in improving adherence and more research is needed to evaluate sustained behavior change over a longer period.	84.6
^37^	Adherence to antihypertensive medications among family practice patients with diabetes mellitus and hypertension	Canada	a cross-sectional sub study	Self-report	Morisky Medication Adherence Scale	77%	N/A	Future studies will need to determine whether focusing adherence strategies on these patients will improve their Cardiovascular outcomes	75
^31^	Adherence to Medication among patients with Hypertension and Diabetes Mellitus in selected Tea Estates in South India.	India	Cross sectional descriptive study	Self-report	Morisky Medication Adherence Scale	76.30%	N/A	Health care accessibility is an important factor which determines adherence to medication especially in chronic diseases like hypertension and diabetes mellitus	62.5
^28^	Community health workers improve disease control and medication adherence among patients with diabetes and/or hypertension in Chiapas, Mexico: an observational stepped-wedge study	Mexico	Cross-sectional study	Self report	Self-reported adherence(survey)	N/A	CHW (Community health workers)	We offer evidence from a prospective study documenting an association between a CHW-led intervention and improved clinical control and medication adherence among patients with diabetes and/or hypertension in a rural Latin American setting.	75
^34^	Medication Adherence and its Association with Glycemic Control, Blood Pressure Control, Glycosuria and Proteinuria Among People Living with Diabetes (PLWD) in the Ho Municipality, Ghana	Ghana	Cross-sectional study	Self-report	Morisky, Green and Levine Adherence Scale	60.67%	N/A	Patient counselling to attain optimal medication adherence should, therefore, be intensified.	75
^32^	Influential Factors in Adherence to the Therapeutic Regime in Hypertension and Diabetes	Columbia	Cross-sectional study	Nursing outcomes classification	Nursing Outcomes Classification	N/A	N/A	Health services providers and for health professionals, it is important to know the factors that influence on the behavior of adherence of individuals with processes of chronic disease, like arterial hypertension and T2DM, given that these affect people's behaviors, leading them to not complying strictly with the therapeutic regime prescribed and, thereby, not complying with the therapeutic objectives	87.5
^43^	Psychosocial and behavioral correlates of self-efficacy in treatment adherence in older patients with comorbid hypertension and type 2 diabetes	Italy and Poland	Cross-sectional study	Adherence to refill medication	MGLS (Morisky Green Levine Scale),AMR(Adherence to refill medication scale),INAS(Intentional non-adherence)	N/A	N/A	Adopting a patient-reported adherence approach, future clinical research and practice may consider these associations in order to develop further empirical assessments and psychosocial and behavioral interventions with the purpose of fostering adherence to clinical prescriptions, and consequently, increasing health-related quality of life of this chronic population.	75
^38^	Evaluation of a hypertension medication therapy management program in patients with diabetes	United states of America	Randomized control trial	Adherence was calculated using the continuous measure of medication acquisition method, in which the days' supply of a medication is compared with the dates the medication is filled	prescription claims data	7%	A community pharmacy–based hypertension MTM program	Community pharmacists are strategically positioned to provide MTM services and effectively communicate with providers to help maximize patient outcomes and improve quality of care.	92.3
^29^	Impact of Socio–Economic, Health and Patient Related Factors on Medication Adherence in Patients with Hypertension and Type II Diabetes	India	Cross-sectional study	Self-report	MMAS8	860(29.86%)	N/A	Healthcare professionals are therefore required to engage chronic patients in order to improve positive health outcomes, communicate with them about their health beliefs, and provide appropriate information about their disease and treatment. This helps both healthcare professionals and patients collaborate effectively.	62.5
^30^	Characteristics of Patients with Primary Non-adherence to Medications for Hypertension, Diabetes, and Lipid Disorders	United states of America	Retrospective Cohort study	proportion of days covered (PDC)	Adherence was calculated using the proportion of days covered (PDC)	7%	N/A	We recommend that healthcare systems pursue directly linking orders with dispensed prescriptions	54.5
^39^	Factors Associated with Antihypertensive Medication Adherence among Diabetic Patients with Coexisting Hypertension in a Tertiary Care Centre from a Low Middle Income South Asian Country	Sri Lanka	Cross-sectional study	Self-report	modified MASES (Medication Adherence Self Efficacy Scale) questionnaire	53.3%(Males) 38.5%(Females)	N/A	Prescribers to consider patients socio demographic factors such as gender, income, and occupation when choosing the appropriate pharmaceutical agents to control hypertension.	75
^44^	Determinants of Treatment Adherence and Health Outcomes in Patients with Type 2 Diabetes and Hypertension in a Low-Income Urban Agglomerate in Delhi, India: A Qualitative Study	India	Qualitative study	Self-report	Morisky, Green and Levine Adherence	73%	N/A	Expanding the role of community health workers or volunteers in the prevention and treatment of NCDs and including information regarding nonpharmacological interventions in health promotion packages might help to improve treatment outcomes, adherence, and patient treatment pathways to care.	60
^13^	Magnitude and associated factors of poor medication adherence among diabetic and hypertensive patients visiting public health facilities in Ethiopia during the COVID-19pandemic	Ethiopia	Cross-sectional study	Self-report	Morisky Medication Adherence Scale	28%	N/A	All concerned health authorities should take into account, and set multidisciplinary strategies to prevent impacts of the COVID-19 pandemic on medication adherence of patients with chronic illnesses.	75
^41^	Medication adherence and its correlates among diabetic and hypertensive patients seeking care from Primary Health Center, India	India	Cross-sectional study	Self-report	Morisky Medication Adherence Scale	16.7% (low adherence)	N/A	Focused health education sessions addressing the importance of adherence to medications need to be carried out regularly	50
^23^	Effectiveness of Home Telehealth in Comorbid Diabetes and Hypertension: A Randomized, Controlled Trial	United states of America	single center, randomized, controlled clinical trial	Self-report	a validated regimen adherence scale	N/A	nurse, physician, and a certified diabetes educator	Further studies are needed to evaluate the optimal frequency and intensity of monitoring of home monitoring with nurse monitoring across all levels of patient risk	84.6

N/A: Not available.

### 2.6 Data charting and synthesis

The following information were extracted from each study using a standardized data extraction form: study title, authors, primary affiliation of author, year, study design, study settings, methods, country of study, study focus, factors affecting adherence (categorized as health-related factors, clinical condition-related factors, therapy-related factors, patient-related factors, and socio-economic factors), adherence measures, tools for medication adherence, study limitations, and recommendations.

### 2.7 Collating, summarizing, and reporting results

The findings were reported following the review questions, including information on prevalence, adherence evaluation, associated factors, and interventions relating to medication adherence in patients with co-morbid hypertension and diabetes. In reporting the data, implications for pharmaceutical care, clinical practice, and policy were also highlighted.

## 3 Results

### 3.1 Study characteristics

The database searches revealed 1123 potentially relevant studies. After removing duplicates, 972 articles were examined. Following the screening of titles and abstracts, 43 full-text papers were chosen. 31 papers were considered for analysis after the eligibility criteria were applied to the full-text articles(Fig. 1). Included among 31 papers were 5 randomized controlled trials/intervention studies, 2 cohort studies, 2 qualitative studies and 22 cross-sectional studies. Regarding the article's country of origin, 7 investigations were conducted in the United States, 5 in India, 4 in Ghana, 3 in Nigeria, 2 in Mexico, and 1 each in Indonesia, Sri Lanka, Canada, Saudi Arabia, Poland, Italy/Poland, Malaysia, Brazil, and Colombia (Table 1).

### 3.2 Factors associated medication adherence in co-morbid hypertension and diabetes

The factors associated with medication adherence in individuals with co-morbid hypertension and diabetes were categorized into health-related factors, condition related factors, therapy related factors, patient related factors and socio-economic related factors, as outlined below (Table 2):

**Table 2:** Description of factors associated with medication adherence in co-morbid hypertension and diabetes.

Study	Health related factors	Condition related factors	Therapy related factors	Patient related factors	Socio-economic factors
^26^	Pharmacist education and counselling	N/A	N/A	N/A	N/A
^35^	N/A	Absence of disease complications	Drug side effect, drug combination	Age	Mode of payment for medical bills
^47^	N/A	N/A	N/A	N/A	Social support
^36^	NA	N/A	More >4 medications have low adherence, patients on insulin therapy	Female and lower education	Health insurance
^24^	Patients who receive care from the same physician for both conditions and prescribe statins frequently	LDL, HDL, and triglycerides	Number of index medications, statin medications	Age (older patients)	N/A
^17^	Poor prescription instruction by health providers	N/A	Number of medications	N/A	Societal norms
^16^	N/A	N/A	High medication related burden	N/A	Monthly expenditure on medications
^25^	Pharmacist	N/A	N/A	N/A	N/A
^33^	N/A	HbA1c	N/A	N/A	N/A
^40^	N/A	N/A	N/A	Female, high sch	unemployed
^42^	N/A	N/A	N/A	emotional response, depressive symptoms	N/A
^45^	N/A	N/A	N/A	Overall perception of quality of life and health score	N/A
^21^	Pharmacist education and counselling	FBS, SBP, DBP, BMI	N/A	N/A	N/A
^14^	N/A	N/A	N/A	Belief about medication	N/A
^48^	N/A	N/A	N/A	N/A	N/A
^46^	N/A	N/A	N/A	N/A	Lack of finance
^27^	Motivational interview-trained pharmacy students	N/A	medication burden	Previous hospitalization.	N/A
^37^	N/A	N/A	Number of prescription medications	Age, sex, education level.	N/A
^31^	Prolong duration of time for consultation	Complications	Frequently changed medications	N/A	N/A
^28^	Community health worker intervention	N/A	N/A	N/A	N/A
^34^	N/A	Glycemic and blood pressure control	N/A	N/A	N/A
^32^	People caring for them respond to their concerns and difficulties with respect to their treatment	N/A	N/A	knowing about their health condition.	economic resources to travel for consultation, Have support from their families or close acquaintances,
^43^	N/A	N/A	N/A	Positive beliefs about medication, stronger perceived medication-specific social support	N/A
^38^	Pharmacist	N/A	Medication Therapy management	None	N/A
^29^	Provider patient relationship, frequency of visits, availability of medicine, medication cost	N/A	number of medicines, side effects, therapy duration	inadequate knowledge about therapy.	social support
^30^	Outpatient clinic visits, phone calls and emails,	N/A	Number of medications	smoking status	insurance product
^39^	N/A	N/A	N/A	Male	low income and employment
^44^	N /A	N/A	N/A	Forgetfulness of therapy	Family support through reminders
^49^	Attendance to a Health center	Presence of comorbidities	N/A	Substance use, level of education	Lack of financial resources (extreme poverty)
^41^	N/A	N/A	N/A	higher education, tobacco use	N/A
^23^	Nurses and physicians involved in home tele intervention and a certified diabetes educator.	N/A	N/A	N/A	N/A

N/A: Not Available.

#### 3.2.1 Health-related factors

Health related factors included the nurses involved in home telehealth intervention,^23^ patients who receive care from the same physician for both conditions and prescribe statins frequently,^24^physician involved in home telehealth intervention,^23^ a certified diabetes educator,^23^pharmacist education and counselling^21^^,^^25^,pharmacist brief telephone intervention,^26^ motivational interview by trained pharmacist student,^27^ community healthcare workers intervention,^28^availability of medicine^29^ out-patient clinic visits,^29^^,^^30^phone calls,^30^ emails,^30^ attendance to health center,^13^ prolong duration of time for consultation,^31^ poor prescription instruction by health providers^17^ Provider patient relationship^29^ and the health care professionals response to their concerns and difficulties respect to their treatment.^32^

#### 3.2.2 Condition-related factors

The condition-related factors included Low Density Lipoprotein(LDL), High Density Lipoprotein(HDL), Triglycerides levels^24^ Fasting blood sugar(FBS), Systolic blood pressure(SBP), Diastolic blood pressure(DBP), Body Mass Index(BMI)(,^21^ glycated hemoglobin(HbA1c),^33^ glycemic and blood pressure control^34^,presence of co-morbidity^13^ and absence of disease complications.^35^

#### 3.2.3 Therapy-related factors

The following therapy-related factors affected medication adherence: the number of medications,^17^^,^^24^^,^^29^^,^^30^^,^^36^^,^^37^ medication-related burden,^16^^,^^27^ medication therapy management,^38^ frequency of medication change,^31^ side effects,^29^^,^^35^ drug combination^35^, therapy duration,^29^ insulin therapy^36^ and statins use.^24^

#### 3.2.4 Patient-related factors

Patient related factors that impacted on medication adherence included sex of patient^36^^,^^37^^,^^39^^,^^40^, age of patient,^24^^,^^35^^,^^37^ level of education,^13^^,^^36^^,^^37^^,^^40^^,^^41^ mental health factors^42^^,^^43^, previous hospitalization^27^, belief of medication^14^^,^^43^,substance and tobacco use,^13^^,^^30^^,^^41^ forgetfulness of therapy^44^,level of knowledge about their health condition and therapy^29^^,^^32^ and perception of quality of life.^45^

#### 3.2.5 Socio-economic related factors

Health insurance,^30^^,^^36^ lack of financial resources^13^^,^^46^, employment status^39^^,^^40^, societal norms,^17^ social support,^29^^,^^32^^,^^47^ monthly expenditure on medications,^16^ Medication Costs^29^,mode of payment for medical bills^35^, low income^39^,economic resources to travel for consultation,^32^ have support from their families or close acquaintances,^32^ family support through reminders^44^ were the socio-economic factors associated with medication adherence among individuals with comorbid hypertension and diabetes.

### 3.3 Medication adherence measuring tools

There were 32 individually different tools used to measure adherence. Likewise, 29 studies used a single adherence measuring tool, 1 study used two different tools, 1 study used three. The majority of studies employed self-reporting tools to assess medication adherence, with 21 studies exclusively relying on self-reporting tools as the sole measure of adherence outcomes. Proportion of days covered was the most frequent objective measurements of adherence (*n* = 5). Adherence levels ranged from as low as 7% to as high as 80%, reflecting the variability in patient adherence.

### 3.4 Intervention types and study outcomes in co-morbid hypertension and diabetes

The various interventions used to improve medication adherence included education and counselling by pharmacists,^21^^,^^25^ telephone motivational intervention^26^^,^^27^, nurse, physician, and a certified diabetes educator counselling,^23^ medication therapy management^38^ and intervention by community health workers.^28^ Five included studies,^21^^,^^25^, 26., 27, 28. reported that their interventions had a substantial positive effect on medication adherence. Nevertheless, 2 of the studies,^23^^,^^38^ did not find any meaningful influence on any of the adherence outcomes that were examined.

## 4 Discussion

Due to the global health significance of co-morbid hypertension and diabetes^2^, this review incorporates papers from across various continents. Asia and America (including North and South America) are the primary contributors to this review, with United States of America^23^^,^^24^^,^^26^^,^^27^^,^^30^^,^^38^^,^^42^and India^29^^,^^31^^,^^41^^,^^44^^,^^46^ contributing the most studies, respectively. The prevalence of medication adherence in patients with co-morbid hypertension and diabetes ranged from 7% to 80%.^26^^,^^30^^,^^38^ These variations could be because of many factors that influence medication adherence and differences in cultural settings that the studies were conducted.^50^ These factors include health care related, therapy related, condition related, patient related and socio-economic factors^51^.

The main health care related factors that influenced medication adherence was the involvement health care professionals in patient care^21^^,^^23^, 24., 25, 26.^,^^38^ and patient relationships with providers.^29^ Inadequate patient-provider relationships can lead to insufficient counselling and negatively affect patients' self-efficacy.^50^ Trust-based communication and effective discussion of adherence issues can improve patient self-efficacy and therapy adherence. Neglecting these factors can worsen disease severity.

In terms of condition related factors co-morbidity^13^^,^^30^^,^^35^ and complications^31^^,^^35^ are significant factors associated with medication adherence, particularly in chronic illnesses like cardiovascular events, kidney failure, and neuropathy. These complications often require complex prescription regimens and lifestyle changes, which can overwhelm patients and lead to non-adherence.^35^ The cumulative impact of managing various health disorders can make it difficult to continuously adhere to recommended treatments.

Co-morbid hypertension and diabetes patients often require more prescribed medications, leading to increased medication-related burden.^16^^,^^20^^,^^52^^,^^53^ To address this, it is crucial to streamline treatment regimens, use combination medicines when suitable, and ensure patients receive sufficient knowledge about their condition and medication.^50^^,^^54^ Effective medication management requires a collaborative approach involving patients and healthcare practitioners.^38^^,^^55^ Adherence to prescribed medication can be influenced by side effects, medication interactions, and duration of therapy. These factors can be improved through continuous patient education, simplification of therapy, and fixed-dose combinations.^22^

Socio-economic factors that influence co-morbid patients' medication adherence is their access to health insurance, employment status, cultural beliefs, and social networks. Insufficient insurance coverage can hinder patients' ability to afford prescribed medications, leading to financial barriers.^56^ Support programs, insurance expansion, and reduced out-of-pocket costs can help alleviate these barriers.^57^ Employment-related obstacles can be mitigated through employer-sponsored health programs. Cultural ideas and societal conventions influence health behaviors, including medication adherence.^58^ The stigma associated with chronic illnesses might impede open dialogues on treatment and discourage the disclosure of such conditions. Promoting patient's adherence can be accomplished by the implementation of educational programs and culturally sensitive healthcare procedures that address these norms and concerns. Social support networks^59^ and emotional encouragement from family, friends, or community groups can improve adherence.

Medication adherence is significantly influenced by patient specific factors such as gender, education level, and age. Healthcare professionals can improve health outcomes in managing chronic conditions like co-morbid hypertension and diabetes by implementing a patient-centered approach.^60^ Personalized educational interventions, clear communication, and regular assessments of patient comprehension are essential. Sustaining adherence requires prioritizing long-term benefits, addressing patient-specific concerns, and cultivating a positive quality of life.^45^^,^^61^

To improve medication adherence in co-morbid hypertension and diabetes, numerous interventions have been implemented by pharmacists and other health care providers around the world.^21^^,^^23^^,^^25^, 26., 27, 28. Most interventions necessitate the patient's participation in decision-making pertaining to their condition.

### 4.1 Strength of the study

This review provides synthesized evidence of the current state of medication adherence studies with focus on the factors and interventions for patients with co-morbid hypertension and diabetes. The review contributes to evidence-based decision making and provides support for areas of practice that require further development.

### 4.2 Limitation of study

Though the current review utilized a transparent and rigorous methodology, it is not devoid of limitations. In this review three databases and one search engine was used for retrieving the documents and this might prevent other potential publications which may not have been indexed in such databases from being missed. Nevertheless, the deliberate omission of non-indexed literature ensured that the selection process was focused solely on peer-reviewed, minimally biased works.

## 5 Conclusion

Significant influences on medication adherence in co-morbid hypertension and diabetes patients were seen in relation to the patient, condition, therapy, socio-economic status, and health-related factors. Interventions designed to enhance adherence in individuals with co-morbid hypertension and diabetes must consider the complex interaction of these factors. The implementation of customized interventions, which involve the cooperation of healthcare professionals, policymakers, and researchers, is critical to optimize medication adherence and, consequently, improve the health outcomes of patients. This review serves as a fundamental basis for formulating focused interventions and encouraging more scholarly investigations in this pivotal healthcare domain. Future studies should delve into the progression of adherence behaviors among individuals who are concurrently treated with hypertension and diabetes. Critical interventions to examine include the integration of digital health solutions, the investigation of shared decision-making processes, and the streamlining of complex medication regimens. Furthermore, gaining insight into the interaction between health policies and patient-reported outcomes will enhance the body of evidence and enable the optimization of treatments to achieve better results for patients who have co-morbid hypertension and diabetes.

## Funding

This research did not receive any specific grant from funding agencies in the public, commercial, or not-for-profit sectors.

## CRediT authorship contribution statement

**Adwoa Oforiwaa Kwakye:** Conceptualization, Data curation, Formal analysis, Writing – original draft. **Irene A. Kretchy:** Conceptualization, Data curation, Formal analysis, Writing – original draft. **Prince Peprah:** Writing – review & editing. **Kofi Boamah Mensah:** Writing – review & editing.

## Declaration of competing interest

The authors declare that they have no known competing financial interests or personal relationships that could have appeared to influence the work reported in this paper.

## Appendix A Supplementary data

Joanna Briggs Institute quality assessment tools for included studies. Supplementary material
