# Supplementary material for: Factors influencing medication adherence in co-morbid hypertension and diabetes patients: A scoping review
Source: Explor Res Clin Soc Pharm. 2024 Feb 29;13:100426. doi: 10.1016/j.rcsop.2024.100426 (PMC10918559; doi:10.1016/j.rcsop.2024.100426)
Supplement: Supplementary file 1 — Joanna Briggs Institute quality assessment tools for included studies. [file mmc1.docx]

**Supplementary Table 1: Critical appraisal results for cross-sectional studies**

| **Citation** | **Q1** | **Q2** | **Q3** | **Q4** | **Q5** | **Q6** | **Q7** | **Q8** | **%** |
| --- | --- | --- | --- | --- | --- | --- | --- | --- | --- |
| (Adenike Bamigbola et al., 2017) | N | Y | Y | Y | Y | N | Y | Y | 75 |
| (Adisa et al., 2017) | Y | Y | Y | Y | N | N | Y | Y | 75 |
| (Akrom & Anggitasari, 2019) | Y | Y | Y | Y | U | N | Y | Y | 75 |
| (An & Nichol, 2013) | Y | Y | Y | Y | Y | Y | Y | Y | 100 |
| (Baah-Nyarkoh et al., 2023) | Y | Y | Y | Y | Y | N | Y | Y | 87.5 |
| (Fadare et al., 2015) | Y | Y | Y | Y | N | N | Y | Y | 75 |
| (Jankowska-Polańska et al., 2021) | Y | Y | Y | Y | N | N | Y | Y | 75 |
| (Keeley & Driscoll, 2013) | N | Y | Y | Y | Y | N | Y | Y | 75 |
| (Khayyat et al., 2019) | Y | Y | Y | Y | N | N | Y | Y | 75 |
| (Lingam et al., 2021) | Y | Y | Y | Y | N | N | Y | Y | 75 |
| (Mazzuchello et al., 2016) | Y | Y | Y | Y | N | N | Y | Y | 75 |
| (Medi et al., 2015) | Y | Y | Y | N | N | N | Y | N | 50 |
| (Natarajan et al., 2013) | Y | Y | Y | Y | N | N | Y | Y | 75 |
| (Navya et al., 2015) | Y | Y | Y | Y | N | N | Y | N | 62.5 |
| (Newman et al., 2018) | Y | Y | Y | Y | N | N | Y | Y | 75 |
| (Osei-Yeboah et al., 2019) | Y | Y | Y | Y | N | N | Y | Y | 75 |
| (Parra et al., 2019) | Y | Y | Y | Y | N | Y | Y | Y | 87.5 |
| (Pierobon et al., 2023) | Y | Y | Y | Y | N | N | Y | Y | 75.0 |
| (Prasad & Kumar, 2021) | Y | Y | Y | Y | N | N | Y | N | 62.5 |
| (Ratnayake et al., 2021) | Y | Y | Y | Y | N | N | Y | Y | 75.0 |
| (Shimels et al., 2021) | Y | Y | Y | Y | N | N | Y | Y | 75.0 |
| (Thekkur et al., 2015) | Y | Y | U | Y | U | U | Y | U | 50 |
|  | 95.4% | 100% | 95.4% | 95.4% | 18% | 9.09% | 100% | 81.8% |  |

Y=Yes, N=no U=Unclear

Q1. Were the criteria for inclusion in the sample clearly defined?

Q2. Were the study subjects and the setting described in detail?

Q3. Was the exposure measured in a valid and reliable way?

Q4. Were objective, standard criteria used for measurement of the condition?

Q5. Were confounding factors identified?

Q6. Were strategies to deal with confounding factors stated?

Q7. Were the outcomes measured in a valid and reliable way?

Q8. Was appropriate statistical analysis used?

**Supplementary Table 2: Critical appraisal results for qualitative studies**

| **Citation** | **Q1** | **Q2** | **Q3** | **Q4** | **Q5** | **Q6** | **Q7** | **Q8** | **Q9** | **Q10** | **%** |
| --- | --- | --- | --- | --- | --- | --- | --- | --- | --- | --- | --- |
| (Atinga et al., 2018) | U | Y | Y | Y | Y | U | U | U | Y | Y | 70 |
| (Sharma et al., 2023) | U | Y | Y | Y | Y | U | U | Y | Y | U | 60 |
|  | 0% | 100% | 100% | 100% | 100% | 0% | 0% | 50% | 100% | 50% |  |

Y=Yes, N=no U=Unclear

Q1. Is there congruity between the stated philosophical perspective and the research methodology?

Q2. Is there congruity between the research methodology and the research question or objectives?

Q3. Is there congruity between the research methodology and the methods used to collect data?

Q4. Is there congruity between the research methodology and the representation and analysis of data?

Q5. Is there congruity between the research methodology and the interpretation of results?

Q6. Is there a statement locating the researcher culturally or theoretically?

Q7. Is the influence of the researcher on the research, and vice- versa, addressed?

Q8. Are participants, and their voices, adequately represented?

Q9. Is the research ethical according to current criteria or, for recent studies, and is there evidence of ethical approval by an appropriate body?

Q10. Do the conclusions drawn in the research report flow from the analysis, or interpretation, of the data?

**Supplementary Table 3: Critical appraisal results for cohort studies**

| **Citation** | **Q1** | **Q2** | **Q3** | **Q4** | **Q5** | **Q6** | **Q7** | **Q8** | **Q9** | **Q10** | **Q11** | **%** |
| --- | --- | --- | --- | --- | --- | --- | --- | --- | --- | --- | --- | --- |
| (Abughosh et al., 2016) | Y | Y | U | Y | U | U | Y | Y | U | U | Y | 54.5 |
| (Raebel et al., 2012) | U | Y | Y | Y | U | U | Y | Y | U | U | Y | 54.5 |
|  | 50% | 100% | 50% | 100% | 0% | 0% | 100% | 100% | 0% | 0% | 100% |  |

Y=Yes, N=no U=Unclear

Q1. Were the two groups similar and recruited from the same population?

Q2. Were the exposures measured similarly to assign people to both exposed and unexposed groups?

Q3. Was the exposure measured in a valid and reliable way?

Q4. Were confounding factors identified?

Q5. Were strategies to deal with confounding factors stated?

Q6. Were the groups/participants free of the outcome at the start of the study (or at the moment of exposure)?

Q7. Were the outcomes measured in a valid and reliable way?

Q8. Was the follow up time reported and sufficient to be long enough for outcomes to occur?

Q9. Was follow up complete, and if not, were the reasons to loss to follow up described and explored?

Q10. Were strategies to address incomplete follow up utilized?

Q11. Was appropriate statistical analysis used?

**Supplementary Table 4: Critical appraisal results for RCT/Intervention study**

| **Citation** | **Q1** | **Q2** | **Q3** | **Q4** | **Q5** | **Q6** | **Q7** | **Q8** | **Q9** | **Q10** | **Q11** | **Q12** | **Q13** | **%** |
| --- | --- | --- | --- | --- | --- | --- | --- | --- | --- | --- | --- | --- | --- | --- |
| (Contreras-Vergara et al., 2022) | y | Y | Y | Y | N | N | Y | Y | Y | Y | Y | Y | Y | 84.6 |
| (Kwakye et al., 2021) | Y | Y | Y | Y | Y | Y | Y | U | Y | Y | Y | Y | U | 84.6 |
| (Mohan et al., 2023) | Y | Y | Y | Y | N | N | Y | Y | Y | Y | Y | Y | N | 84.6 |
| (Planas et al., 2009) | Y | Y | Y | Y | N | Y | Y | Y | Y | Y | Y | Y | Y | 92.3 |
| (Wakefield et al., 2011) | Y | Y | Y | Y | N | Y | Y | Y | Y | Y | Y | Y | N | 84.6 |
|  | 100% | 100% | 100% | 100% | 20% | 60% | 100% | 100% | 100% | 100% | 100% | 100% | 40% |  |

Y=Yes, N=no U=Unclear

Q1. Was true randomization used for assignment of participants to treatment groups?

Q2. Was allocation to treatment groups concealed?

Q3. Were treatment groups similar at the baseline?

Q4. Were participants blind to treatment assignment?

Q5. Were those delivering treatment blind to treatment assignment?

Q6. Were outcomes assessors blind to treatment assignment?

Q7. Were treatments groups treated identically other than the intervention of interest?

Q8. Was follow up complete and if not, were differences between groups in terms of their follow up adequately described and analyzed?

Q9. Were participants analysed in the groups to which they were randomized?

Q10. Were outcomes measured in the same way for treatment groups?

Q11. Were outcomes measured in a reliable way?

Q12. Was appropriate statistical analysis used?

Q13. Was the trial design appropriate, and any deviations from the standard RCT design (individual randomization, parallel groups) accounted for in the conduct and analysis of the trial?
